# Identification of novel amino acid variants in the Han Chinese population that impair toll-like receptor 4 signaling and confer hyporesponsiveness to lipopolysaccharide

**DOI:** 10.3389/fimmu.2025.1556600

**Published:** 2025-10-01

**Authors:** Bin Zhou, Zhaoying Lv, Ruijuan Zu, Jianbo Liu, Yuting Yang, Zongguang Zhou, Yuan Li

**Affiliations:** ^1^ Laboratory of Digestive Surgery, State Key Laboratory of Biotherapy and Cancer Center, West China Hospital, West China School of Medicine, Sichuan University, Chengdu, Sichuan, China; ^2^ Division of Gastrointestinal Surgery, Department of General Surgery, West China Hospital, Sichuan University, Chengdu, Sichuan, China

**Keywords:** toll-like receptor 4, missense variant, severe acute pancreatitis, NF-kappa B, lipopolysaccharide

## Abstract

**Introduction:**

Severe acute pancreatitis (SAP), characterized by life-threatening inflammation and multiorgan failure, involves Toll-like receptor 4 (TLR4)-mediated hyperinflammation. This study investigates TLR4 variants’ impact on disease severity.

**Methods:**

Sanger sequencing was performed in a Han Chinese cohort of patients with acute pancreatitis to screen for *TLR4* variants. In silico analyses predicted structural consequences of identified missense variants on protein conformation, transmembrane domains, and isoelectric points. Co-immunoprecipitation assays assessed interactions between wild-type or mutant TLR4 and MyD88 in human embryonic kidney (HEK) 293T cells. Luciferase reporter assays and interleukin 8 (IL-8) expression tests evaluated nuclear factor kappa B (NF-κB) activation and downstream inflammatory signaling. To validate clinical relevance, knock-in mice carrying the *Tlr4* p.(Arg804Trp) variant were generated, and SAP models were established to compare histopathological scores, apoptosis, immune cell infiltration, and serum biomarkers (amylase, lipase, IL-6, IL-10, tumor necrosis factor alpha) between homozygous mutant and wild-type controls.

**Results:**

Five novel missense variants in *TLR4* were identified in the Han Chinese cohort. In silico analyses predicted subtle alterations to the protein’s secondary structure for all five variants, with p.(Arg763Cys) specifically affecting the transmembrane domain and theoretical isoelectric point. Co-immunoprecipitation revealed that p.(Gly715Ser), p.(Arg763Cys), and p.(Arg804Trp) failed to interact with MyD88. Functional characterization in HEK293T cells confirmed that these three variants suppressed NF-κB activation and downstream IL-8 expression. In knock-in mice, homozygous *Tlr4* p.(Arg804Trp) mutants exhibited significantly lower histological scores, reduced apoptosis, decreased neutrophil and macrophage infiltration, and lower serum levels of amylase, lipase, IL-6, and IL-10 compared to wild-type controls.

**Couclusion:**

We characterized novel *TLR4* variants that compromise its signaling function, resulting in lipopolysaccharide hyporesponsiveness and attenuated SAP. These findings underscore the significance of genetic determinants in modulating SAP severity and advocate for TLR4-targeted interventions as a promising therapeutic approach.

## Introduction

Acute pancreatitis (AP) is a prevalent and potentially life-threatening condition, with significant variability in its severity (1). Approximately 20% of patients with AP progress to severe acute pancreatitis (SAP), a condition linked to a mortality rate between 20% and 40% (2). Despite advancements in the diagnosis and treatment of AP, there has been no substantial decline in mortality rates over the past decade (3).

Our understanding of the progression of AP is still remarkably limited, leaving therapeutic interventions largely confined to conservative supportive measures (4). In the vast majority of cases, acute pancreatitis is a noninfectious inflammatory condition, also known as sterile inflammation, that affects the pancreas. Over the past decade, activation of the innate immune system has emerged as a critical trigger, amplifier, and effector of sterile inflammatory injury in solid organs. This activation significantly influences the degree of parenchymal cell death, remote organ damage, and disease resolution (5).

Toll-like receptor 4 (TLR4) is a principal receptor that recognizes lipopolysaccharide (LPS) derived from gram-negative bacteria and plays a pivotal role in the innate immune system (6). The engagement of TLR4 triggers the activation of nuclear factor kappa B (NF-κB) through the myeloid differentiation primary response gene 88 (Myd88)-dependent pathway, culminating in the transcription of pro-inflammatory cytokines such as tumor necrosis factor alpha (TNFα), interleukin 1 beta (IL-1β), interleukin 6 (IL-6), and interleukin 8 (IL-8). Additionally, TLR4 can elicit an alternative, Myd88-independent pathway by stimulating the TIR domain-containing adapter molecule 1 (TRIF)-mediated activation of interferon regulatory factors (IRFs), leading to the production of interferons (IFNs) (7). In recent years, a growing body of research has linked TLR4 to a broad spectrum of diseases, including acute pancreatitis, cardiovascular disease, Alzheimer’s disease, diabetes, inflammatory bowel disease and liver-related disease (8–13).

Two TLR4 missense variants, specifically p.(Asp299Gly) and p.(Thr399Ile), are known to co-segregate among individuals of European ancestry, leading to a reduced responsiveness to endotoxins and an association with a variety of infectious and non-infectious diseases (14–22). In contrast, these variants are virtually absent in Asian populations (23–26). In our study, we examined the incidence and distribution of TLR4 polymorphisms within the Han Chinese population. We identified five novel missense variants in the human TLR4 gene and further analyzed their potential roles in the pathogenesis of SAP.

## Materials and methods

### Subjects

This study adhered to the principles set forth in the Declaration of Helsinki and was granted approval by the Ethical Review Board of West China Hospital, Sichuan University (Reference number: 2018172A). A total of 338 unrelated patients with acute pancreatitis (AP) were enrolled, of which 206 suffered from severe acute pancreatitis (SAP). Furthermore, 524 unrelated individuals with no clinical or biochemical evidence of AP, matched for age and sex, were selected as controls following routine health examinations at the hospital. All patients and control subjects were recruited from West China Hospital, Sichuan University. Prior to their inclusion in the study, all participants or their legal representatives provided informed consent.

### DNA amplification and sequencing

DNA was extracted from whole blood samples. Two overlapping fragments of the TLR4 exon 4 were polymerase chain reaction (PCR)-amplified by the following primers: TLR41F (5′-CTATTTTAGGTTCTTATTCAGCAGAA-3′), TLR41R (5 ′-GAATACTGAAAACTCACTCATTTGTT-3′), TLR42F (5′-gtttcaaaggttgctgttctcaa-3′), and TLR42R (5′-CTTATTAACTGAACAAGTGTTGGA-3′). All amplifiers were generated using PCR in a PTC-100 thermocycler (MJ Research, Waltham, MA). Reactions were performed in 50 µL containing 1.5mM MgCl_2_, 1× buffer, 0.25mM each dNTP, 0.32µM each primer,2 U Taq polymerase or PrimeSTAR HS DNA polymerase (Takara, Da Lian, PR China). Amplification conditions were 35 cycles of 95°C for 30 s, 54°C for 60 s, and 72°C for 90 s. PCR products were assessed on an agarose gel, purified, and sequenced. An ABI 3730 DNA analyzer (Applied Biosystems Inc., Foster City, CA) was used for sequencing, and the results were analyzed using the GeneTool Lite 1.0 (BioTools Inc., Edmonton, Alberta, Canada) software program.

### *In silico* analysis

The secondary structures of wild-type TLR4 and its mutants were predicted using the Self-Optimized Prediction Method with Alignment (SOPMA) tool, available at https://npsa-pbil.ibcp.fr/cgi-bin/npsa_automat.pl?page=/NPSA/npsa_sopma.html. The transmembrane domains of wild-type and variant TLR4 proteins were identified using the SOSUI algorithm (https://harrier.nagahama-i-bio.ac.jp/sosui/). The theoretical isoelectric point of both wild-type TLR4 and its mutants was determined using the Compute pI/Mw tool, accessible at https://web.expasy.org/compute_pi/.

### Expression plasmid construction and site-directed mutagenesis of human TLR4

The expression plasmids encoding human TLR4 in vectors GV219 (untagged) or GV366 (HA tagged) and human Myd88 in CV702 (Flag tagged)were purchased from GeneChem Corporation (Shanghai, PR China). Expression plasmids encoding untagged human CD14 and MD-2 in the vector pCMV6-XL5 were procured from OriGene Technologies (Wuxi, PR China). NF-κB vector and pRL-TK reporter plasmids were supplied by Promega Corporation (Madison, WI). The c.2143G>A(p.(Gly715Ser)), c.A2247T(p.(Phe749Leu)), c.2287C>T(p.(Arg763Cys)), c.2410C>T(p.(Arg804Trp)), and c.2467C>A(p.(Pro823Thr)) mutant plasmids based on TLR4-GV219 or TLR4-GV366 were developed by PCR site-directed mutagenesis with primers. The plasmids were then treated with DpnI (New England biolabs, Ipswich, MA) to eliminate the parental methylations from the newly synthesized unmethylated mutant DNA and transformed into *Escherichia coli* cells where the nick was ligated by host repair enzymes (27). The outcomes of site-directed mutagenesis were ultimately validated by direct sequencing.

### Co-immunoprecipitation assay

HEK 293T cells were a kind gift from Dr. ChuanWen Fan (Sichuan University, Sichuan, PR China) and authenticated using the Short Tandem Repeat (STR) genotyping method. HEK 293T cells were cultured in DMEM supplemented with 10% FBS, 2 mM L-glutamine, 100 units/mL penicillin and 100 µg/mL streptomycin. All cells were maintained in a 5% saturated CO_2_ atmosphere at 37°C. For co-immunoprecipitation, HEK293T cells were seeded into 10 cm tissue culture dishes and co-transfected with 8 µg TLR4 WT-HA or five variants-HA and 8 µg Myd88-Flag plasmids using Turbofect transfection reagent (Thermo Fisher Scientific, Waltham, MA). Two days post-transfection, cells were harvested and lysed with a cold lysis buffer (200 µl/well) containing 50 mM Tris (pH 7.4), 150 mM NaCl, 1% Triton X-100, 0.5% sodium deoxycholate, 0.1% SDS, 1× protease inhibitor cocktail on ice for 30 min. The lysates were incubated over night with nano HA-Trap magnetic beads (Proteintech, Wuhan, P.R. China) or nano DYKDDDDK(flag)-Trap magnetic beads (Proteintech).Binding Control magnetic beads or 293T blank cells were used as controls. After overnight rotation, the magnetic beads were washed three times with 500 µl/tube ice pre-cooled lysis buffer, following which 80 ml of 2× loading buffer was used to elute the proteins which were mixed gently. The processed samples were run on an SDS gel, transferred to PVDF membrane, and the primary Ab of HA(Abclone, Wuhan, P.R. China) or DDK(Origene, Beijing, P.R. China) were used to detect HA or Flag labelled content in the IP products.

### Transient transfection, IL-8 assay and NF-КB luciferase reporter assay

HEK 293T Cells were plated to maintain a density of 1.5*10^5^ per well in 12-well dishes and incubated overnight. The following day, the cells were transiently co-transfected with optimized quantities of GV219-TLR4 or its mutants (300 ng/well), MD-2 vector (3 ng/well) and CD14 vector (30 ng/well), together with NF-κB vector (500 ng/well) and pRL-TK reporter plasmid (100 ng/well) using Turbofect transfection reagent (28). After 24 h, cells were stimulated with LPS (5 ng/mL) for another 24 h. After stimulation, the supernatants were collected and analyzed for IL-8 with a Luminex kit (R&D Inc., Meriden, CT) on Luminex 100, and the remaining cells were washed with PBS and lysed to detect luciferase activity. Luciferase activity was determined using a luciferase kit, following the manufacturer’s instructions (Promega). Firefly luciferase activity was normalized to Renilla luciferase activity. Values in cells transfected with TLR4 or its mutants were normalized to GV219 empty transfected cells and expressed as fold induction. The results depicted the mean of triplicate determinations ± SD.

### Western blotting

Whole cell lysates from HEK293T cells were prepared using a cold lysis buffer (200 µl/well) containing 50 mM Tris (pH 7.4), 150 mM NaCl, 1% Triton X-100, 0.5% sodium deoxycholate, 0.1% SDS, 1× protease inhibitor cocktail on ice for 30 min. The cell lysate concentrations were measured using an enhanced bicinchoninic acid (BCA) protein assay kit (thermo). Proteins (20 mg) were fractionated in 12% SDS-PAGE and transferred to a polyvinylidene fluoride membrane (Merck Millipore,Darmstadt, Germany).The membranes were blocked with 5% non-fat dry milk and probed with the appropriate primary antibodies diluted in Tris-buffered saline with Tween-20 (TBST) including TLR4(1:1000),NF-κB p65 (1:1000), CD14 (1:1000), MD2 (1:1000) and β-actin (1:1000) at 4°C overnight. All above primary antibodies were purchased from Abclone. The membranes were washed four times with TBST (10 min/time) and incubated with horseradish peroxidase-conjugated anti-mouse IgG (CST) or horseradish peroxidase-conjugated anti-rabbit IgG (CST). The blots were visualized using a chemiluminescence detection kit (Biosharp, He Fei, P.R. China). Semi-quantitative analysis of protein expression levels was conducted by densitometry using Image Lab software (Bio-Rad, Hercules, CA).

### Animals

All experimental procedures were sanctioned by the Animal Research Committee of Sichuan University (Reference number: 20211433). Genetically modified mice carrying the TLR4 p.(Arg804Trp) point mutation were obtained from Cyagen Biosciences (Suzhou, P.R. China), generated using CRISPR/Cas-mediated genome editing in C57BL/6 mice. Genotyping was performed by extracting DNA from tail samples, followed by PCR amplification and sequencing. Male C57BL/6 mice homozygous for the p.(Arg804Trp) variant, along with their wild-type (WT) littermates, aged 6-8 weeks and weighing 22-24 grams, were selected for the study. All mice were maintained at the Experimental Animal Center of West China Hospital. The animals were kept in a controlled environment with access to standard rodent chow and water ad libitum. They underwent a 12-hour fast prior to experimentation, with water available freely throughout. All mice used in experiments were handled according to the guidelines and approved protocols of the Cedars-Sinai Medical.

Center Animal Care and Use Committees (IACUC protocol # 9214).

### Induction of SAP

The induction of SAP was carried out in accordance with the methods described by Ding et al. and Li et al. (29, 30). Both p.(Arg804Trp) mice and their wild-type counterparts received seven hourly injections of cerulein (50 µg/kg in a total volume of 0.2 mL), with LPS(10 mg/kg) being administered concurrently with the final cerulein injection. Mice from all groups serving as controls were given normal saline (0.2 mL) at equivalent time points. All animals underwent euthanasia 18 hours after the last injection, followed by removal of the pancreas for histological evaluation using hematoxylin and eosin staining. Blood samples were centrifuged (1000g, 5 minutes) to obtain serum, which was subsequently analyzed for levels of amylase, lipase, and the cytokines tumor necrosis factor alpha (TNFα), interleukin 6 (IL-6), and interleukin 10 (IL-10).

### Morphologic examination, TUNEL and immunofluorescence assay

Mice were euthanized, and liver tissues were harvested and fixed in 4% paraformaldehyde. Then the tissues were embedded in paraffin and sectioned at 5-mm intervals. Hematoxylin and eosin (H&E) staining was performed according to standard procedures. Multiple randomly selected microscopic fields from at least five mice per group were evaluated and scored by two pathologists who were blinded to the experimental conditions. The scoring was based on the presence of vacuolization, interstitial edema, interstitial inflammation, and acinar cell necrosis, as previously established (31). Each parameter was graded on a scale of 0 to 3 (with 0 indicating normal and 3 indicating severe changes), and the cumulative score was used to determine the severity of severe acute pancreatitis. Apoptosis was assessed by TUNEL bright red apoptosis detection kit (Vazyme, Nanjing, China) according to the manufacturer’s instructions. For immunofluorescence assay, sections were stained with antibodies rat anti-mouse F4/80 [(Abclone) and rabbit anti-mouse LY6G (Abclone) at 4°C overnight. Followed by incubation with a secondary antibody(FITC-conjugated Goat anti-Rat IgG (H+L) or ABflo^®^ 594-conjugated Goat anti-Rabbit IgG (H+L), respectively]. Finally, sections of TUNEL or immunofluorescence assay were mounted with antifade mountants with DAPI (thermo)before imaging with Vectra Polaris multispectral imaging system (PerkinElmer) with 20× objective. For TUNEL or immunofluorescence assays, cellular analysis was performed using the ‘Analyze Particles’ plugin of ImageJ2 software (National Institutes of Health). At least 5 non-overlapping fields per well were captured and analyzed. Data are presented as the mean ± SD from at least three independent biological replicates. The proportion of positive cells was determined by dividing the number of positive cells by the total cell count, which was represented by DAPI staining. An average of 500 ~1000 cells were counted across all fields within each replicate.

### Assays (amylase, lipase, IL-6, IL-10, TNFα,TLR4)

Serum amylase and lipase levels were quantified using an automated biochemical analyzer (Beckman Coulter, Miami, FL). The serum concentrations of cytokines IL-6, IL-10, and TNFα were measured with Luminex assay kits following the manufacturer’s protocol (R&D Systems) on a Luminex 200 system (Austin, TX). Digital images of the bead arrays were acquired after laser excitation and subsequently analyzed using a computer workstation.

For Western blot analysis, pancreatic tissues were a commercial lysis buffer (Epizyme Biotech, Shanghai, P.R. China) supplemented with a protease inhibitor cocktail (Selleck Chemicals, Houston, TX), and homogenized using a dispersion machine (IKA, Staufen, Germany). Protein concentrations were determined using a bicinchoninic acid protein assay kit (Thermo Fisher Scientific). A total of 30 μg of protein samples were separated by SDS-PAGE, transferred to a PVDF membrane, blocked with 5% non-fat milk in TBST containing 0.1% Tween 20, and incubated with mouse monoclonal antibody against mouse TLR4 (Proteintech, Wuhan, P.R. China) or rabbit monoclonal antibody against mouse β-actin (CST, Danvers, MA).

### Statistics analysis

The results are presented as mean ± standard error of the mean (S.E.M.). Data were analyzed using one-way analysis of variance (ANOVA) followed by *post hoc* multiple comparison tests. Statistical significance was considered at P < 0.05. All statistical analyses were conducted using SPSS version 17 for Windows (SPSS Inc., Chicago, IL).

## Results

### Variant analysis

**Figure 1:**
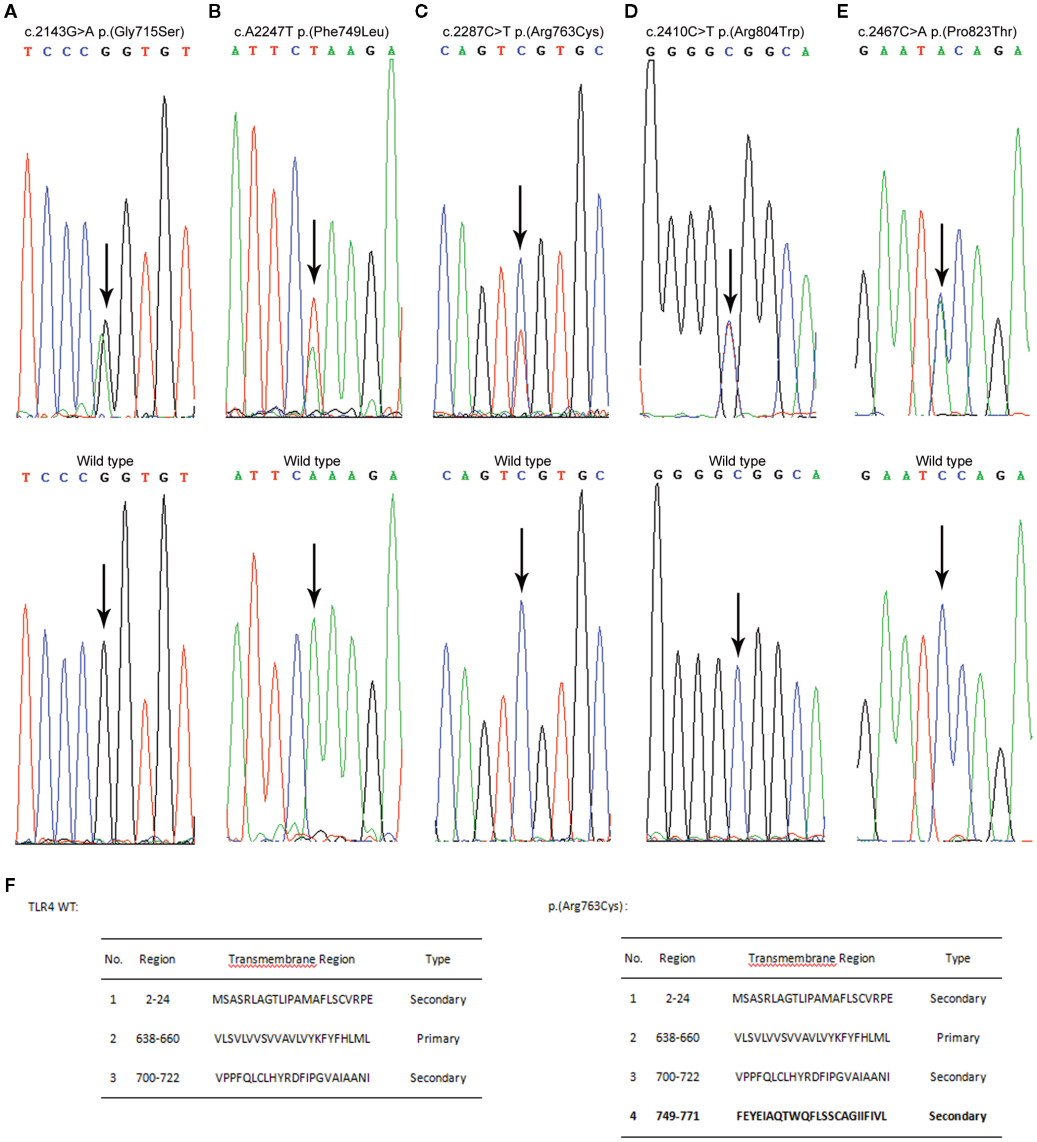
Variant analysis results are presented alongside the wild-type sequences for comparative purposes (shown in the lower panels). The nucleotide substitutions are indicated by arrows, and the concomitant changes at the amino acid level are documented within parentheses. **(A)** Direct sequencing reveals a heterozygous G to A transition at nucleotide position 2143. **(B)** An arrow indicates a heterozygous A to T transversion at nucleotide position 2247. **(C)** An arrow denotes a heterozygous C to T transition at nucleotide position 2287. **(D)** An arrow signifies a heterozygous C to T transition at nucleotide position 2410. **(E)** An arrow points out a heterozygous C to A transversion at nucleotide position 2467. **(F)** Transmembrane domain prediction was performed with SOSUI (https://harrier.nagahama-i-bio.ac.jp/sosui/). Experiments were repeated at least twice.

We used sequence analysis to predominantly screen the coding region of TLR4 in 338 AP patients and 524 age- and sex-matched healthy individuals. No p.(Asp299Gly) or p.(Thr399Ile) variants were detected in any of the subjects. Nonetheless, we identified five novel amino acid variants (**Figure 1**) and submitted the information to the LOVD database (https://www.lovd.nl/). All five variants were localized in the TIR (Toll-IL-1-Receptor) domain but were not co-segregated. The distribution of TLR4 variants was in Hardy–Weinberg equilibrium (*P*>0.05) in both the study and control populations, but the minimum allele frequency in both the patient and control groups is less than 1% (**Table 1**). The p.(Phe749Leu) and p.(Arg804Trp) were detected only in patients group. Interestingly, all patients with amino acid variants suffered from SAP (**Table 2**).

**Table 1:** Results of screening and genotyping for *TLR4* gene in the Chinese population.

Nucleotide position	C.a896g	C.c1196t	C.2143g>a	C.2247t>a	C.2287c>t	C.2410c>t	C.2467c>a
Amino acid change	p.(Asp299Gly)	p.(Thr399Ile)	p.(Gly715Ser)	p.(Phe749Leu)	p.(Arg763Cys)	p.(Arg804Trp)	p.(Pro823Thr)
LOVD accession numbers	#0000251826	#0000314527	#0000734302	#0000935647	#0000935648	#0000929510	#0000735281
Number of heterozygotes of patients with acute pancreatitis(n=338)	0	0	2	1	1	1	4
Minor allele frequency in patients with acute pancreatitis	0	0	0.0029	0.0015	0.0015	0.0015	0.0059
Number of heterozygotes in normal population (n=524)	0	0	3	0	1	0	2
Minor allele frequency in normal population	0	0	0.0029	0	0.00095	0	0.0019

**Table 2:** Clinical characteristics of the patients and molecular characteristic of *TLR4* variants.

Patient	1	2	3	4	5	6	7	8	9
Sex	male	male	male	female	female	female	female	female	female
Age	40	66	37	37	68	38	41	34	51
Classification of AP	SAP	SAP	SAP	SAP	SAP	SAP	SAP	SAP	SAP
Other related disease	Chronic cholecystitis	Lung infection, multiple organ failure, shock	Chronic superficial gastritis	gallstone	Chronic superficial gastritis	Hyperlipidemia	Cholecystitis, Chronic superficial gastritis	Chronic cholecystitis	Gastric multiple ulcer
DNA change	c.2143G>A	c.2143G>A	c.2247T>A	c.2287C>T	c.2410C>T	c.2467C>A	c.2467C>A	c.2467C>A	c.2467C>A
Protein change	p.(Gly715Ser)	p.(Gly715Ser)	p.(Phe749Leu)	p.(Arg763Cys)	p.(Arg804Trp)	p.(Pro823Thr)	p.(Pro823Thr)	p.(Pro823Thr)	p.(Pro823Thr)

### *In silico* analysis

**Figure 2:**
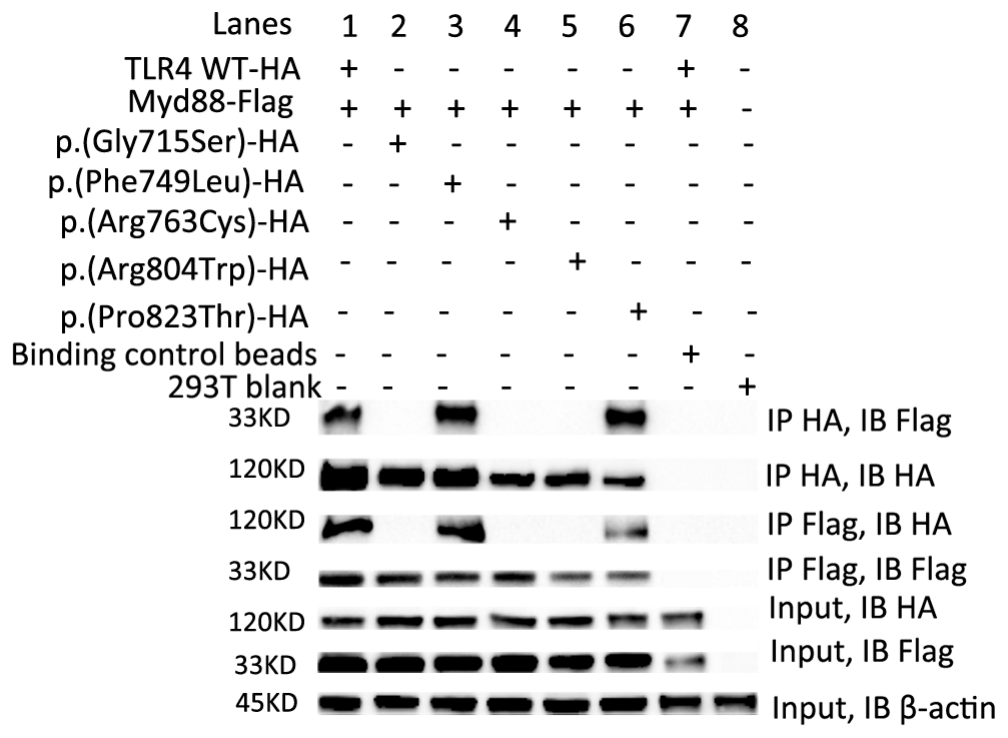
The p.(Gly715Ser), p.(Arg763Cys) and p.(Arg804Trp) failed to interact with Myd88. Transient transfections in HEK293T cells with 8 µg TLR4 WT-HA (lane 1) or five variants-HA (lane 2-6) and 8 µg Myd88-Flag plasmids followed by immunoprecipitation (IP) with HA-Trap magnetic beads or DYKDDDDK (flag)-Trap magnetic beads and analyzed by Immunoblotting (IB) with anti-HA or anti-Flag Ab. Binding Control magnetic beads (lane 7) or 293T blank cells (lane 8) were used as controls. Representative experiment of at least two independent experiments.

Using SOPMA software, we predicted that the secondary structures of these five variant sites differed slightly from those of wild-type TLR4 (**Table 3**). We also discovered a significant difference between the transmembrane domains of p.(Arg763Cys) and TLR4 WT using two transmembrane domain prediction software packages SOSUI (**Figure 2**). Furthermore, the theoretical isoelectric points of TLR4 WT and p.(Arg763Cys) were calculated to be 5.88 and 5.83 using the Compute pI/Mw tool-ProtParam (https://web.expasy.org/protparam/). The theoretical isoelectric points of p.(Gly715Ser), p.(Phe749Leu), p.(Arg804Trp), and p.(Pro823Thr) evaluated by ProtPram is nearly equal to that of TLR4 WT.

**Table 3:** Protein secondary structures were predicted using SOPMA (https://npsa-pbil.ibcp.fr/cgi-bin/npsa_automat.pl?page=/NPSA/npsa_sopma.html).

Prediction of secondary structure	TLR4 WT	P.(gly715ser)	P.(phe749leu)	P.(arg763cys)	P.(arg804trp)	P.(pro823thr)
Alpha helix (Hh)	428 is 51.01%	407 is 48.51%	415 is 49.46%	407 is 48.51%	427 is 50.89%	390 is 46.48%
3_10_ helix (Gg)	0 is 0.00%	0 is 0.00%	0 is 0.00%	0 is 0.00%	0 is 0.00%	0 is 0.00%
Pi helix (Ii)	0 is 0.00%	0 is 0.00%	0 is 0.00%	0 is 0.00%	0 is 0.00%	0 is 0.00%
Beta bridge (Bb)	0 is 0.00%	0 is 0.00%	0 is 0.00%	0 is 0.00%	0 is 0.00%	0 is 0.00%
Extended strand (Ee)	114 is 13.59%	113 is 13.47%	110 is 13.11%	121 is 14.42%	108 is 12.87%	110 is 13.11%
Beta turn (Tt)	24 is 2.86%	18 is 2.15%	23 is 2.74%	20 is 2.38%	18 is 2.15%	19 is 2.26%
Bend region (Ss)	0 is 0.00%	0 is 0.00%	0 is 0.00%	0 is 0.00%	0 is 0.00%	0 is 0.00%
Random coil (Cc)	273 is 32.54%	301 is 35.88%	291 is 34.68%	291 is 34.68%	286 is 34.09%	320 is 38.14%
Ambiguous states ()?	0 is 0.00%	0 is 0.00%	0 is 0.00%	0 is 0.00%	0 is 0.00%	0 is 0.00%
Other states	0 is 0.00%	0 is 0.00%	0 is 0.00%	0 is 0.00%	0 is 0.00%	0 is 0.00%

### The p.(Gly715Ser), p.(Arg763Cys) and p.(Arg804Trp) variants failed to interact with Myd88

Recruitment of Myd88 to TLR4 is one of the earliest events of TLR4 signaling (7).To determine the impact of these five variants on this process, transient transfections in HEK293T cells followed by immunoprecipitation assays were carried out. Our results showed that TLR4 WT, p.(Phe749Leu) and p.(Pro823Thr) could interact with Myd88, while p.(Gly715Ser), p.(Arg763Cys) and p.(Arg804Trp) failed to interact with Myd88 (**Figure 2**).

### The p.(Gly715Ser), p.(Arg763Cys) and p.(Arg804Trp) variants impaired TLR4-mediated activation

**Figure 3:**
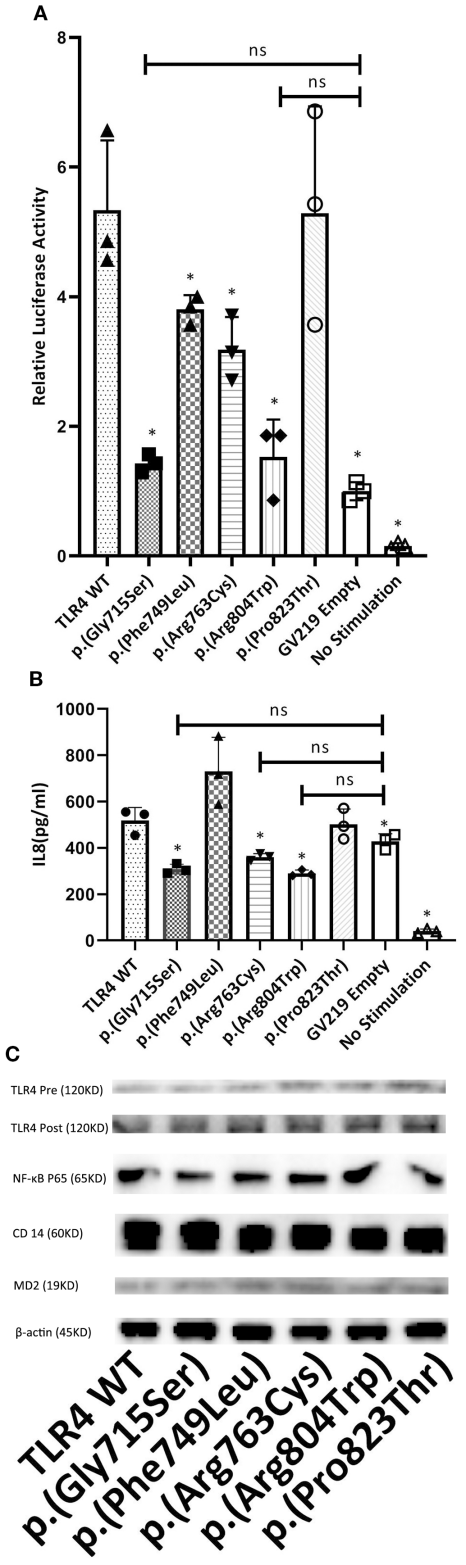
The study investigated the effect of TLR4 variants on LPS-induced NF-*K*B signaling and IL-8 production in HEK 293T cells. Cells were co-transfected with optimal amounts of GV219-TLR4 or its variant forms (p.(Gly715Ser), p.(Phe749Leu), p.(Arg763Cys), p.(Arg804Trp), p.(Pro823Thr)) at 300 ng/well, along with MD-2 (3 ng/well), CD14 (30 ng/well), NF-*K*B vectors (500 ng/well), and the pRL-TK reporter plasmid (100 ng/well). Following a 24-hour transfection period, cells were challenged with LPS at a concentration of 5 ng/mL for an additional 24 hours. **(A)** To assess the impact on signaling activity, cell lysates were assayed for firefly and renilla luciferase activities. The firefly luciferase activity was normalized against renilla luciferase activity. The results from cells transfected with TLR4 or its mutants were further normalized against those from cells transfected with the empty GV219 vector and are presented as fold induction. **(B)** For IL-8 quantification, supernatants were collected after the LPS treatment and analyzed using a Luminex kit (R&D Systems) on the Luminex 100 platform. **(C)** The expression of TLR4 before (Pre) and after (Post) transfection, along with that of NF-κB p65, CD14, MD-2, and β-actin, is shown in representative western blots for each genotype. The data shown represent the mean ± SD from triplicate measurements performed in three independent experiments **(A, B)**. *P<0.05, compared with TLR4 WT. Ns, no significance. ANOVA and Dunnett’s test.

As all the identified variants were localized in the cytoplasmic TLR4 TIR region, they could potentially affect LPS signaling and TLR4-mediated activation. To test this hypothesis, human embryonic kidney (HEK) 293T cells were transfected with pRL-TK reporter plasmids encoding engineered WT and mutant TLR4 (untagged), CD14, MD-2 (to impart LPS sensitivity), and NF-κB proteins. Then, we performed the dual luciferase reporter assay to analyze NF-κB activity and a Luminex assay to quantify Il-8-cytokines associated with TLR4 downstream signal transduction through a Myd88-dependent pathway that could amplify a cascade of inflammatory reactions (32). The dual luciferase reporter assay results depicted that, in comparison to WT TLR4, the NF-κB activities decreased drastically in p.(Gly715Ser) and p.(Arg804Trp) (approximately 70%, *P* < 0.05) and moderately in p.(Phe749Leu) and p.(Arg763Cys) (approximately 20–40%, *P* < 0.05) (**Figure 3A**). There were no significant differences among p.(Gly715Ser), p.(Arg804Trp), and the GV219 empty group(*P*>0.05). Our results demonstrated that IL-8 levels of p.(Gly715Ser), p.(Arg763Cys), and p.(Arg804Trp) were considerably lower than WT TLR4 (approximately 40%, *P* < 0.05) and the GV219 empty group (**Figure 3B**). Consequently, these results elucidated that the p.(Gly715Ser),the p.(Arg763Cys) and the p.(Arg804Trp) variants impaired TLR4-mediated activation. The expression levels of TLR4, MD2, CD14, and NF-kB reporter were aligned in each group confirmed by Western blotting (**Figure 3C**).

### The p.(Arg804Trp) variant mouse models of cerulean- and LPS-induced pancreatitis exhibited endotoxin hyporesponsiveness and lower severity of SAP

**Figure 4:**
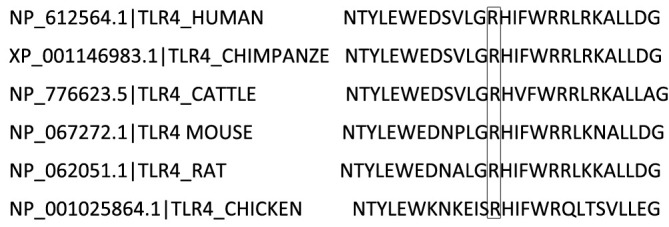
Species-specific comparison of a segment within the TLR4 protein sequence is illustrated. The residue at position 804, Arginine (R), which is subject to variation, is emphasized with a box.

**Figure 5:**
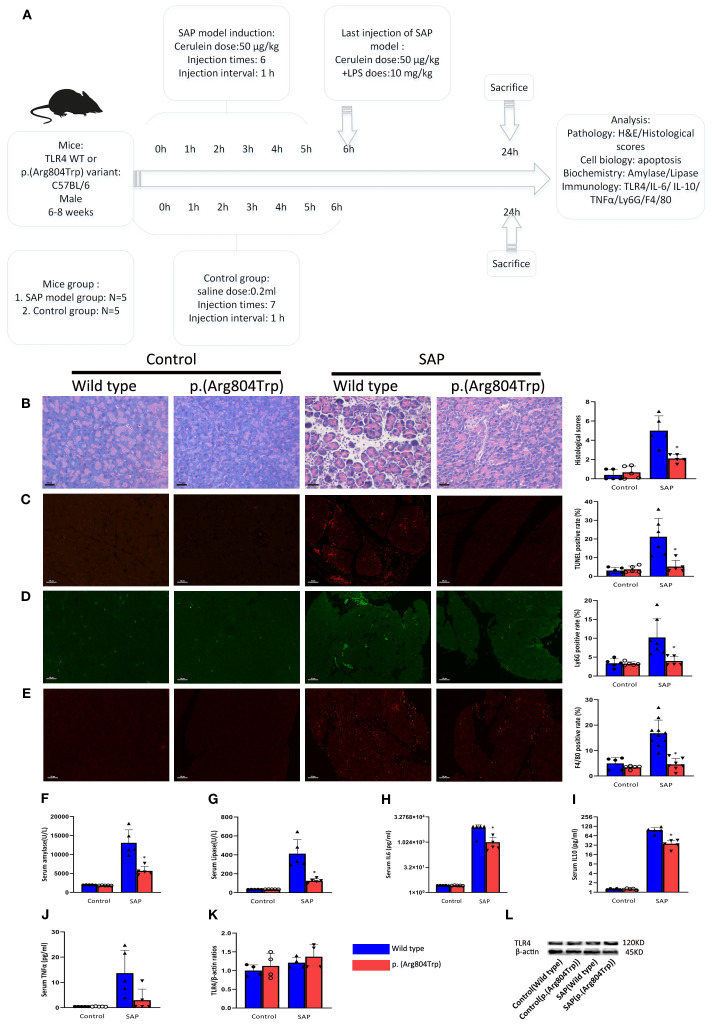
Compared to TLR4 WT mice, the SAP-modeled p.(Arg804Trp) mice exhibited reduced responsiveness to endotoxin. **(A)** Overview of SAP mice model induced by cerulean and LPS and design of the experiment. **(B)** Representative images of pancreatic morphological changes and histologic scores in the SAP models are shown. Hematoxylin and eosin-stained histological sections of the pancreas from WT and p.(Arg804Trp) mice were selected. **(C)** Representative images and quantitative analysis of TUNEL staining on pancreas sections from TLR4 WT or p.(Arg804Trp) mice with saline or SAP modeling. **(D, E)** Representative images and quantitative analysis of neutrophil(Ly6G) and macrophage markers(F4/80) on pancreas sections from TLR4 WT or p.(Arg804Trp) mice with saline or SAP modeling. The ratios of positive cells was determined by dividing the number of positive cells by the total cell count represented by DAPI staining. The images were magnified 200×. Serum amylase **(F)** and lipase **(G)** levels were measured using an automatic biochemical analyzer. Cytokine levels of IL-6 **(H)**, IL-10 **(I)**, and TNFα **(J)** in serum were quantified using Luminex assay kits on the Luminex 200 system. **(K)** The average densitometric ratios of TLR4 to β-actin for each group are illustrated. **(L)** Representative western blots show TLR4 and β-actin expression in each group.* denote a statistically significant difference (*P* < 0.05) between the p.(Arg804Trp) group and the wild type group in SAP modeling by ANOVA and Dunnett’s test. Data represent one experiment representative of at least three independent trials.

Given the ~66% identity between mouse and human TLR4 protein sequences and the conservation of the p.(Arg804Trp) mutation (**Figure 4**), we generated murine models of SAP harboring this variant to investigate its functional role. Histological analyses confirmed the successful generation of typical SAP in the murine model. **Figure 5A** illustrates the induction of SAP in mice using cerulean and LPS, and outlines the experimental design for the subsequent study. Relative to TLR4 WT controls, p.(Arg804Trp) mutant mice displayed significant reductions in histological severity (**Figure 5B**), apoptosis (**Figure 5C**), neutrophil and macrophage infiltration (Ly6G & F4/80; **Figures 5D–E**), and serum amylase, lipase, IL-6, and IL-10 levels (**Figures 5F–I**) (*P* < 0.05 for all comparisons), but not in TNF-α expression (**Figure 5J**). Furthermore, we performed western blotting to compare the TLR4 protein expression levels in each group (**Figure 5L**). Densitometry analysis revealed that TLR4 expression in the SAP model groups (TLR4 wild-type and p.(Arg804Trp) variant) was only marginally higher than in the normal saline control group, although this difference did not reach statistical significance (**Figure 5K**). These findings suggest that the p.(Arg804Trp) variant is linked to endotoxin hyporesponsiveness and attenuated severity of cerulean- and LPS-induced SAP in mice.

## Discussion

TLR4 is an essential receptor that responds to LPS constituting gram-negative bacterial endotoxin. In the present study, we screened *TLR4* genes in a Han Chinese population and identified five novel missense variants. These variants were not co-segregated, exhibited relatively low minor allele frequencies, and were speculated to be spontaneous in nature. Intriguingly, these variants were all localized in the TIR domain of TLR4 and all patients with amino acid variants suffered from SAP. The TIR domain is located in the intracellular region of TLR4 molecules and is the functional region where TLR4 molecules bind to downstream molecules (33, 34).

We employed a suite of in silico software tools to anticipate the potential effects of these new variants on the structural stability of TLR4. Subsequently, we engineered TLR4 WT-HA or five variants-HA and Myd88-Flag plasmids and using them into co-IP assay. Moreover, TLR4 WT or five variants along with an NF-κB reporter construct and additional plasmids were co-transfected into HEK 293T cells. Following transfection, the cells were stimulated with LPS. We then monitored the activity of downstream NF-κB as well as the expression of the key cytokine IL-8 in HEK 293T cells to assess the activation status of the TLR4 signaling pathway. We selected the HEK-293T cell line over other types, such as pancreatic acinar cells, due to its superior co-transfection efficiency and capacity to reliably detect differences among TLR4 variants. Although our HEK-293T clones constitutively expressed endogenous TLR4, we carefully monitored its expression levels both before and after transfection to mitigate any confounding effects. Moreover, it is important to note that HEK 293T cells express only a limited range of cytokines, such as IL-8 (35–38). This limitation is consistent with our experimental results, where we did not detect the expression of IL-1β, IL-6, and TNFα(data not shown) except for IL-8, in the Luminex experiment. Variant details are discussed in the following:

The variant p.(Gly715Ser) failed to interact with Myd88, exhibited a significant reduction in both NF-κB activity and IL-8 levels compared to the TLR4 wild type, yet displayed no substantial differences when benchmarked against the GV219 empty vector control group. Therefore, we concluded that variant p.(Gly715Ser) is capable of blocking TLR4 signaling.

The variant p.(Phe749Leu) could still bind to Myd88, displayed a modest decrease in NF-κB activity compared to the TLR4 wild type, while its IL-8 levels were slightly elevated. The underlying cause for this discrepancy remains unclear.

The variant p.(Arg763Cys) was specifically predicted to modify the transmembrane domain of TLR4 and theoretical isoelectric points, as determined by SOSUI and ProtParam, respectively. Co-IP analyses revealed that this phenotype fails to associate with Myd88. Both its NF-κB activity and IL-8 levels were mildly reduced in comparison with the TLR4 wild type. Taken together, these findings lead us to conclude that this mutation blocks the TLR4 signaling pathway.

For the variant p.(Arg804Trp), results from Co-IP assays demonstrated no interaction between this phenotype and MyD88.The p.(Arg804Trp) exhibited a substantial reduction in both NF-κB activity and IL-8 levels compared to the TLR4 wild type. Due to resource constraints, the p.(Arg804Trp) site was selectively targeted for point mutation to create murine models of SAP. Notably, histological scores, apoptosis, neutrophil counts, macrophage counts, serum levels of amylase, lipase, IL-6, and IL-10 were significantly reduced in p.(Arg804Trp) mice compared to wild-type (WT) mice, while the TLR4/β-actin ratios remained relatively consistent across different groups. In summary, it is likely that the p.(Arg804Trp) variant is linked to endotoxin hyporesponsiveness in SAP modeled mice mainly through alterations in spatial conformation, rather than affecting TLR4 expression.

The variant p.(Pro823Thr) was the most frequently observed among these five variants, identified in four SAP patients and two healthy controls. However, it maintains robust interaction with MyD88, its NF-κB activity and IL-8 levels did not significantly differ from those of the TLR4 wild type. As a result, we concluded that the p.(Pro823Thr) variant does not appear to influence TLR4 signaling.

As for the p.(Asp299Gly) and p.(Thr399Ile) variants, which are common among individuals of European ancestry, they have been shown to lead to a reduced responsiveness to endotoxins. However, they do not appear to be associated with the severity of AP in several reports (39, 40). We believe this may be due to the different positions of these variants on the TLR4 molecule, the p.(Asp299Gly) and p.(Thr399Ile) variants were localized in the extracellular domain of TLR4 (14, 29).

Our research validates that genetic elements could impact the intensity of acute pancreatitis, and inhibiting TLR4 maybe anticipated to diminish the severity of SAP. Both TLR4 knock-out mice and C3H/HeJ(p.(Pro712His)) mice in SAP models also showed ameliorated severity of AP, compared with TLR4 wild-type group (6, 8, 41). Furthermore, there are a number of reports regarding the use of compounds (artesunate, biochanin A, baicalein, picroside II, quercetin) or herbs from traditional Chinese medicine (Chaiqin chengqi decoction) or treatment (carbo monoxide-based therapy, early continuous blood purification) or biomacromolecule (amuc_1100) or probiotics (*Bifidobacterium*) through TLR4 inhibition, which could alleviate the severity of AP (42–51). This indicates that TLR4 is a promising target for the treatment of SAP.

In summary, our study comprehensively investigates the role of five novel missense variants within the TLR4 gene in a Chinese cohort. Three of these variants can block TLR4 signaling. One specifically confers endotoxin hyporesponsiveness and protects against SAP in mice, evidenced by reduced disease severity. Our findings confirm that genetic determinants modulate acute pancreatitis severity, and TLR4 inhibition holds promise as a therapeutic strategy to mitigate SAP.

## Data Availability

The original contributions presented in the study are included in the article/supplementary files, further inquiries can be directed to the corresponding author/s.
